# Distinct position-specific sequence features of hexa-peptides that form amyloid-fibrils: application to discriminate between amyloid fibril and amorphous β-aggregate forming peptide sequences

**DOI:** 10.1186/1471-2105-14-S8-S6

**Published:** 2013-05-09

**Authors:** A Mary Thangakani, Sandeep Kumar, D Velmurugan, M Michael Gromiha

**Affiliations:** 1 Department of Crystallography and Biophysics, University of Madras, Chennai 600025, India; 2 Biotherapeutics Pharmaceutical Sciences, Pfizer Inc., MC6S, 575 Maryville Centre Drive, St. Louis, MO 63141, USA; 3 Department of Biotechnology, Indian Institute of Technology Madras, Chennai 600036, India

## Abstract

**Background:**

Comparison of short peptides which form amyloid-fibrils with their homologues that may form amorphous β-aggregates but not fibrils, can aid development of novel amyloid-containing nanomaterials with well defined morphologies and characteristics. The knowledge gained from the comparative analysis could also be applied towards identifying potential aggregation prone regions in proteins, which are important for biotechnology applications or have been implicated in neurodegenerative diseases. In this work we have systematically analyzed a set of 139 amyloid-fibril hexa-peptides along with a highly homologous set of 168 hexa-peptides that do not form amyloid fibrils for their position-wise as well as overall amino acid compositions and averages of 49 selected amino acid properties.

**Results:**

Amyloid-fibril forming peptides show distinct preferences and avoidances for amino acid residues to occur at each of the six positions. As expected, the amyloid fibril peptides are also more hydrophobic than non-amyloid peptides. We have used the results of this analysis to develop statistical potential energy values for the 20 amino acid residues to occur at each of the six different positions in the hexa-peptides. The distribution of the potential energy values in 139 amyloid and 168 non-amyloid fibrils are distinct and the amyloid-fibril peptides tend to be more stable (lower total potential energy values) than non-amyloid peptides. The average frequency of occurrence of these peptides with lower than specific cutoff energies at different positions is 72% and 50%, respectively. The potential energy values were used to devise a statistical discriminator to distinguish between amyloid-fibril and non-amyloid peptides. Our method could identify the amyloid-fibril forming hexa-peptides to an accuracy of 89%. On the other hand, the accuracy of identifying non-amyloid peptides was only 54%. Further attempts were made to improve the prediction accuracy *via *machine learning. This resulted in an overall accuracy of 82.7% with the sensitivity and specificity of 81.3% and 83.9%, respectively, in 10-fold cross-validation method.

**Conclusions:**

Amyloid-fibril forming hexa-peptides show position specific sequence features that are different from those which may form amorphous β-aggregates. These positional preferences are found to be important features for discriminating amyloid-fibril forming peptides from their homologues that don't form amyloid-fibrils.

## Background

Outcome of the competition between functionally active and inactive aggregated forms is critical to a protein's fate *in vivo *and *in vitro*. Indeed, aggregation is an ancient threat to proper folding of proteins and it must be overcome by proteins from all organisms to maintain their native functional states. The aggregation of endogenous proteins causes several diseases in humans and animals. Aggregation is also a major hurdle in successful development of biopharmaceutical drug products [1]. In converse biotechnology applications, creation of protein and peptide aggregates with well defined morphologies is of interest for development of nano-materials with desired characteristics.

**Figure 1:**
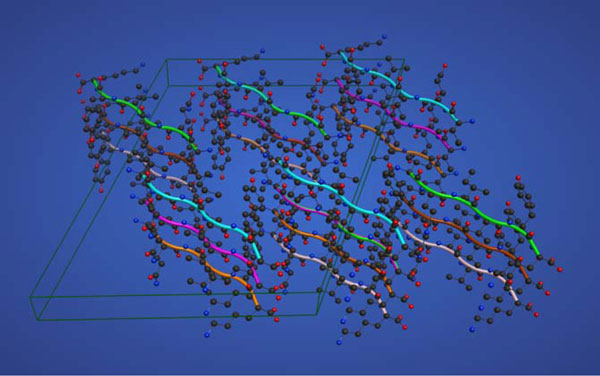
**Microcrystal structure of an amyloid-fibril formed by the hexa-peptide, VQIVYK from a human protein, tau**. The heavy atoms in all the residues are shown in ball and stick representation. Each ribbon represents a hexa-peptide and the box denotes an unit cell.

Plaques containing amyloid fibrils are a common form of protein aggregates that have been detected in several neurodegenerative diseases, such as Alzheimers' [2,3]. These fibrils contain cross-β motif, which yields characteristic reflection in fiber X-diffraction studies [4-6]. This motif arises from short 5-9 residues long sequence regions known as Aggregation Prone Regions (APRs) [7]. The molecular features of this cross-β motif were elucidated by Eisenberg and co-workers [8,9]. Figure 1 illustrates the experimentally known structure of an amyloid-fibril formed by the hexa-peptide, VQIVYK, which is part of the dataset used in this study. Experiments from several research groups have also traced origins of amyloid formation in proteins to short peptide sequences. In particular, Serrano's group has derived amyloidogenic hexa-peptide patterns at neutral and acidic pH by examining the variants of a *de novo *designed amyloid-fibril forming hexa-peptide, STVIIE [10]. Maurer-Stroh et al. [11] have also used amyloid-fibril forming hexa-peptides to develop position specific matrices for prediction of APRs in protein sequences. In order to understand the mechanisms by which proteins are converted from their soluble states to amyloid fibrils, it is essential to analyze the characteristic features of amyloid-fibril forming peptides and compare them with those of the peptides that do not yield amyloid-fibrils under the same experimental conditions but may form amorphous β-aggregates.

On the computational point of view, amino acid properties such as hydrophobicity, β-strand propensity, charge and solubility of amyloid forming peptides have been analyzed and used to predict change in aggregation rate upon mutation [12-14]. Further, several structure-based models and empirical equations have been proposed to predict aggregation prone regions and change in aggregation propensity/rate due to mutation [11,15-21]. Agrawal et al. [1] and Belli et al. [22] have reviewed several commonly available aggregation prediction tools and discussed their advantages and shortcomings towards different applications.

In this work, we have collected and analyzed 139 amyloid-fibril forming hexa-peptides from the experiments of Lopez de La Paz and Serrano [10] and Maurer-Stroh et al. [11]. One hundred and sixty eight hexa-peptide sequences that do not form amyloid-fibrils in experiments conducted by the above mentioned groups were also used. For simplicity, we refer to the two sets as amyloid peptides and non-amyloid peptides. The hexa-peptides in the two datasets are highly homologous as an amyloid peptide may differ from its non-amyloid cousin by just one residue. This indicates that the sequence-structural-thermodynamic features separating amyloid peptides from non-amyloid ones are subtle. Availability of these hexa-peptide sequences with experimental data has afforded us an opportunity to uncover these subtle differences in a systematic manner. The analyses were carried out for several parameters such as overall amino acid composition of the hexa-peptides, preferences for different amino acid residues to occur at each of the six positions in the hexa-peptides and 49 diverse amino acid properties (http://www.cbrc.jp/~gromiha/fold_rate/property.html; [23,24]). Furthermore, we have developed a set of energy potentials based on the propensity of the 20 amino acid residues at six different positions. We found that amyloid peptides show distinct preferences and avoidances for amino acid residues to occur at each of the six positions. These preferences are significantly different from those seen in non-amyloid peptides. Further, we derived energy potentials based on amino acid preferences at different positions in the hexa-peptides and attempted to use them for discriminating amyloid peptides from non-amyloid ones. The success rate for the energy potentials developed to identify amyloid peptides was 89%. This rate compares favorably with that of position specific matrices based program, Waltz, (67%) [11], the structure based program, 3Dprofile (80%) [20], an energy potential based program, PASTA (80%) [25] and statistical mechanics based program, Tango (91%) [10]. On the other hand, the accuracy for negative prediction, that is, prediction of non-amyloid peptides was only 54%. As far as we know, previous studies have not attempted negative predictions and there is no data to compare this negative prediction rate. Further, we have utilized several machine learning algorithms and the method based on random forest discriminated the amyloid and non-amyloid peptides with an accuracy of 82.7% which is a balance between sensitivity (81.3%) and specificity (83.9%).

## Materials and methods

### Collection of amyloid peptide and non-amyloid peptide datasets

We have searched the literature as well as the datasets used in previous works to construct a reliable dataset containing peptide sequences that have been studied experimentally for amyloid-fibril formation. For the purpose of this study, we restricted to hexa-peptide sequences verified with experimental data. This procedure yielded a majority of data from Waltz [11] and Amylhex [10]. In addition, we have used the data reported in the supplementary material of Maurer-Stroh et al. [11]. After eliminating the redundant data, our final dataset contains 139 amyloid forming peptides and 168 non-amyloid peptides.

### Amino acid composition

We have computed the amino acid composition of all the amyloid and non-amyloid peptides using the ratio between the number of amino acids of each type and the total number of residues. It is defined as [26]:

(1) $$\text{Comp}\left(\text{i}\right)=\Sigma {\text{n}}_{\text{i}}/\text{N}$$

where i stands for the 20 amino acid residues. n_i _is the number of residues of each type and N is the total number of residues. The summation is through all the residues in all the considered peptides.

We have also computed the composition of amino acid residues at different positions of the considered hexa-peptides such as position 1, 2, 3, 4, 5 and 6 using the following equation:

(2) $$\text{Comp}\left(\text{i,j}\right)=\Sigma \text{n}\left(\text{i},\text{j}\right)/\text{N}\left(\text{j}\right)$$

where, i and j represent 20 amino acid residues and 6 positions, respectively. N(j) is the total number of residues at position j (i.e. 139 for amyloid and 168 for non-amyloid).

### Position specific amino acid propensities

We have converted the composition of amino acid residues at different positions of hexa-peptides into propensities by normalizing the composition with different factors such as (i) the overall composition of their respective amyloid and non-amyloid forming peptides (Equation 1), β-strand propensity of globular proteins [27] and overall composition of globular proteins [26,28]. After careful inspection of the results we have chosen the propensity based on the normalization with the composition of 20 amino acid residues in globular proteins. The propensity of amino acid residues at different positions is given by

(3) $$\text{Propen}\left(\text{i},\text{j}\right)=\text{Comp}\left(\text{i},\text{j}\right)/\text{Com}{\text{p}}_{\text{glob}}\left(\text{i}\right)$$

where, Comp_glob_(i) is the composition of residue i obtained with a set of globular proteins [26,28]. The values are Ala: 8.47; Asp: 5.97; Cys: 1.39; Glu: 6.32; Phe: 3.91; Gly: 7.82; His: 2.26; Ile: 5.71; Lys: 5.76; Leu: 8.48; Met: 2.21; Asn: 4.54; Pro: 4.63; Gln: 3.82; Arg: 4.93; Ser: 5.94; Thr: 5.79; Val: 7.02; Trp: 1.44 and Tyr: 3.58.

### Energy potentials

The amino acid propensities to occur at each of position of amyloid and non-amyloid peptides were treated as partition functions and converted into thermodynamic energy potential by using the following expression:

(4) $$\phi \left(\text{i},\text{j}\right)=-\text{RT}\;\text{ln}\;\text{propen}\left(\text{i},\text{j}\right)$$

where, i and j are the 20 amino acid residues and six positions respectively.

### Amino acid properties

In this work, we used a set of 49 diverse amino acid properties (physical, chemical, energetic and conformational). These properties have been used in several studies for understanding protein stability, transition state structures of proteins, and predicting protein folding and unfolding rates, discrimination of transporters and structure-function relationship in membrane proteins [29-35]. The numerical values for all the 49 properties used in this study along with their brief descriptions have been explained in our earlier article [23,24] and are freely available at http://www.cbrc.jp/~gromiha/fold_rate/property.html. Besides these properties, we also used a hydrophobicity scale based on retention times of individual amino acids in hydrophobic RPLC columns to compute total hydrophobicity values (H_T_) for each hexa-peptide. This scale is different from all others as it measures latent hydrophobicity of each amino acid [36]. The hydrophobicity coefficient values (h) for each amino acid are as follows: Ala, 13.26; Cys, 26.84; Asp, 8.15; Glu, 11.12; Phe, 90.17; Gly, 3.80; His, 4.14; Ile, 69.53; Lys, 2.92; Leu, 73.84; Met, 51.64; Asn, 1.00; Pro, 27.54; Gln, 6.00; Arg, 10.24; Ser, 3.53; Thr, 11.64; Val, 44.60; Trp, 100.00; Tyr, 47.49; data taken from Table II in [36]. The H_T _value for each hexa-peptide was calculated by summing the hydrophobicity coefficients of the amino acid residues in the hexa-peptide.

### Computation of total amino acid property

The total amino acid property for each hexa-peptide has been computed using the standard formula [37],

(5) $${\text{P}}_{\text{total}}\left(\text{i}\right)=\Sigma \text{P}\left(\text{i},\text{j}\right)$$

where, P(i,j) is the property value of j^th ^residue for the i^th ^peptide and the summation is over 6, the total number of residues in a hexa-peptide. We have repeated the computations for all the 49 amino acid properties in the dataset of amyloid and non-amyloid peptides and the difference between them.

### Discrimination of amyloid and non-amyloid peptides using statistically derived energy potentials

We have made an attempt to discriminate the amyloid and non-amyloid peptides using the energy potentials derived in this work. For this purpose, we combined both amyloid and non-amyloid peptide sets to obtain a set of 307 hexa-peptides. For each hexa-peptide, k in this set, the energy potentials ϕ(i,j) were computed based on propensity value of the i^th ^amino acid ( i = 1, 20) to occur at the j^th ^position (j = 1, 6) as described above. The total potential of the peptide k (ϕ_tot_(k)), was computed by summing over the ϕ(i,j) values for the peptide.

(6) $$\phi_{\text{tot}}\left(\text{k}\right)=\Sigma \phi \left(\text{i},\text{j}\right)$$

These calculations were performed using the potentials derived from both amyloid and non-amyloid peptide sets. The discriminator was then computed as follows:

(7) $$\Delta \phi \left(\text{k}\right)=\phi_{\text{tot}}{\left(\text{k}\right)}_{\text{amyloid}}-\phi_{\text{tot}}{\left(\text{k}\right)}_{\text{non}-\text{amyloid}}$$

If Δϕ (k) has negative value, the peptide is predicted to form amyloid fibrils. Otherwise, it is predicted not to form amyloid-fibrils.

### Machine learning techniques for discriminating amyloid and non-amyloid peptides

We have analyzed several machine learning techniques implemented in WEKA program [38] for discriminating between amyloid and non-amyloid peptides. WEKA includes several methods based on different machine learning techniques such as Bayesian function, Neural network, Radial basis function network, Logistic function, Support vector machine, Regression analysis, Nearest neighbor, Meta learning, Decision tree and Rules. The details of all these methods are available in our earlier articles [37]. We have used the energy potentials and selected amino acid properties as input features for the methods.

### Assessment of predictive ability

We have performed 20-fold, 10-fold and 5-fold cross-validation tests for assessing the validity of the present work. In this method, the data set is divided into n groups, n-1 of them are used for training and the rest is used for testing the method. The same procedure is repeated for n times so that each data is used at least once in the test.

We have used different measures, such as sensitivity, specificity and accuracy, to assess the performance of machine learning methods towards discriminating between amyloid and non-amyloid peptides. The term sensitivity shows the correct prediction of amyloid peptides, specificity is the correct prediction of non-amyloid peptides and accuracy indicates the overall assessment. These terms are defined as follows:

Sensitivity = TP/(TP+FN)

Specificity = TN/(TN+FP)

Accuracy = (TP+TN)/(TP+TN+FP+FN),

where, TP (amyloid peptides predicted as amyloid peptides), FP (non-amyloid peptides predicted as amyloid peptides), TN (non-amyloid peptides predicted as non-amyloid peptides) and FN (amyloid peptides predicted as non-amyloid peptides) refer to the number of true positives, false positives, true negatives and false negatives, respectively.

## Results

### Amino acid composition at different positions of amyloid and non-amyloid peptides

**Figure 2:**
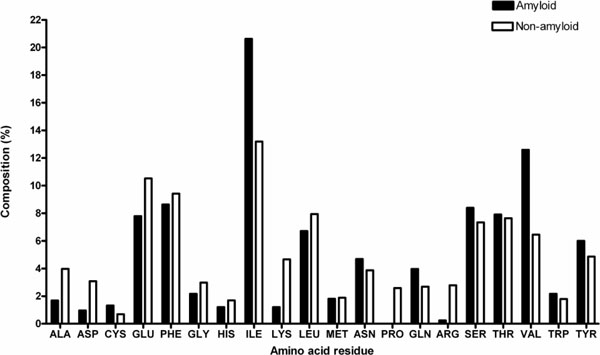
**Amino acid composition in amyloid and non-amyloid forming peptides**.

Amyloid and non-amyloid peptides have different amino acid compositions. Figure 2 compares the overall amino acid compositions of amyloid and non-amyloid peptides. As compared to the non-amyloid peptides, amyloid peptides contain greater proportions of Cys, Ile, Asn, Gln, Ser, Val and Tyr. On the other hand, the non-amyloid peptides contain greater proportions of Ala, Asp, Glu, Phe, Gly, His, Lys, Leu, Pro and Arg. Proportions of Met, Thr and Trp are similar between amyloid and non-amyloid peptides. We noticed that β-branched residues, Ile and Val are considerably more frequent in amyloid peptides whereas the charged residues, Asp, Glu, Lys and Arg, showed considerably higher incidence in non-amyloid peptides than amyloid peptides. These observations are consistent with early observations on composition of amyloidogenic sequences [13,14,39,40].

Amyoid and non-amyloid peptides show different position specific amino acid preferences. We have computed the propensities of amino acid residues to occur at different positions of the amyloid and non-amyloid hexa-peptides. Table 1 shows the preferred and avoided residues at each of the six positions in amyloid and non-amyloid hexa-peptides. An amino acid residue which occurs with a propensity value ≥1.2 at a given position is considered to be preferred at that position. Similarly, the amino acid residue that occurs with a propensity value ≤ 0.8 at a position is considered to be avoided at that position. It can be seen that each position in amyloid or non-amyloid hexa-peptides prefers and avoids different sets of amino acid acids. At 5 of the six positions in the hexa-peptides, the overall position specific preferences for amyloid and non-amyloid peptides are different, although some residues are common (shown in bold in Table 1). At position 5, both amyloid and non-amyloid peptides have the preference for the same residues. Similarly, there are several common residues that are avoided at same positions in both amyloid and non-amyloid peptides.

**Table 1:** Preferred and avoided residues at different positions of amyloid and non-amyloid forming hexa-peptides

Position	Amyloid	Non-amyloid
**Preferred**		
**1**	**S, W, Y, H**, M, N	F, **H**, K, **S, W, Y**
**2**	C, F, I, Q, **T, Y**	E, M, **T, Y**
**3**	**F**, Q, **V, W**	C, E, **F**, N, **V, W**
**4**	**I, L**, N, W, **Y**	F, **I, L, Y**
**5**	**F, I, Y**	**F, I, Y**
**6**	C, **E, F, M**, Q, S, Y	**E, F, M**, T, W
**Avoided**		
**1**	D, T, R, E	G, L, A, Q, V
**2**	**D**, A, **N**, S, G	**D, N**, P, R
**3**	**A, G**, D, I, **Y**	**A, G**, R, T, **Y**
**4**	**V**, S, K, **A**	**A**, M, **V**, D, W
**5**	E, L, Q, **S, T**	A, G, M, R, **S, T**
**6**	**A**, D, G, I, K	**A**, V

Bold letters indicate common amino acid residues in amyloid and non-amyloid.

The difference between amyloid and non-amyloid peptides lies in composition of their core positions. The six positions in the hexa-peptides can be divided into two groups consisting termini (positions 1 and 6) and core (positions 2,3,4 and 5). Most of the preferred residues at the core positions in the amyloid peptides are aromatic or aliphatic. The polar amino acids, Asn and Gln are also preferred at positions 2,3 and 4 of amyloid peptides. In contrast, core positions in non-amyloid peptides can contain charged residues also. At the core positions, several avoided residues are common between amyloid and non-amyloid peptides (Table 1). Taken together, the above observations have revealed the differences between amyloid and non-amyloid peptides and suggested that it may be feasible to discriminate between them at the sequence level via computational means. However, the amino acid composition biases seem to suggest that it may be easier to predict that a given hexa-peptide *will *from amyloid-fibrils than to predict that the given hexa-peptide *will not *from amyloid fibril (see below). This observation is consistent with the view that amyloid-fibril formation is a backbone-driven process [41].

### Energy potentials derived to amino acid propensities

To facilitate the discrimination between amyloid and non-amyloid peptides, we have computed the energy potentials for each amino acid residue to occur at different positions in hexa-peptides and the results are presented in Table 2. We have analyzed the results on two directions: (i) based on amino acid residues and (ii) based on positions. The data presented in Table 2 showed that in amyloids Ser is preferred in position 1; Thr in position 2; Val in position 3; Ile in positions 4 and 5, and Glu in position 6 [42]. The respective preference of residues is lower in the non-amyloid peptides.

**Table 2:** Energy potentials (kcal/mole) computed from the propensities of amino acid residues at different positions in amyloid and non-amyloid hexa-peptides

Amyloid				Position		
	1	2	3	4	5	6
**Amyloid**						
Ala	0.510	0.814	1.469	1.056	0.000	0.814
Asp	0.848	0.848	1.259	0.000	0.000	0.606
Cys	0.000	-0.434	-0.021	-0.021	0.000	-0.263
Glu	0.639	0.335	0.135	0.468	1.294	-0.974
Phe	-0.060	-0.231	-0.833	-0.060	-0.806	-0.300
Gly	-0.007	0.596	1.422	0.000	0.000	1.009
His	-0.386	0.027	0.000	0.681	0.000	0.000
Ile	0.408	-0.137	1.235	-1.225	-1.460	0.580
Lys	0.080	0.000	0.000	1.239	0.000	0.826
Leu	0.310	-0.103	0.231	-0.252	1.056	0.310
Met	-0.291	0.255	0.668	0.000	0.000	-0.491
Asn	-0.142	0.685	0.442	-0.688	0.000	0.029
Pro	0.000	0.000	0.000	0.000	0.000	0.000
Gln	0.340	-0.314	-0.165	0.000	0.996	-0.534
Arg	0.734	0.000	0.000	0.000	0.000	0.000
Ser	-1.036	0.602	0.098	1.259	1.259	-0.171
Thr	0.829	-1.089	0.000	0.175	1.244	0.083
Val	-0.014	-0.071	-1.191	1.355	0.000	-0.071
Trp	-0.413	0.000	-0.546	-0.747	0.413	0.413
Tyr	-0.204	-0.353	0.956	-0.283	-0.798	-0.112
**Non-amyloid**						
Ala	0.423	-0.070	0.622	0.927	0.622	0.757
Asp	0.214	0.718	0.306	0.548	0.414	0.306
Cys	0.000	0.093	-0.320	0.500	0.000	0.000
Glu	-0.073	-0.206	-0.244	-0.021	0.340	-0.874
Phe	-0.250	0.054	-0.772	-0.492	-0.864	-0.359
Gly	0.710	0.375	0.879	0.467	1.528	0.225
His	-0.165	0.139	0.382	0.139	0.382	0.382
Ile	0.280	0.108	0.280	-0.866	-1.122	0.038
Lys	-0.370	0.285	0.192	0.393	0.285	0.393
Leu	0.516	-0.031	0.273	-0.423	0.423	0.011
Met	0.126	-0.178	0.369	0.778	0.778	-0.378
Asn	-0.028	1.207	-0.270	-0.099	0.251	0.385
Pro	0.397	0.566	0.397	0.262	0.262	0.262
Gln	0.451	0.451	0.040	0.282	0.282	-0.052
Arg	-0.050	0.604	0.604	0.300	0.848	0.192
Ser	-0.827	0.132	0.132	0.303	0.716	0.061
Thr	0.117	-0.899	0.530	0.196	0.942	-0.125
Val	0.402	0.310	-0.681	0.645	0.310	0.645
Trp	-0.130	0.114	-0.634	0.521	0.114	-0.130
Tyr	-0.240	-0.360	0.413	-0.170	-0.503	0.243

**Figure 3:**
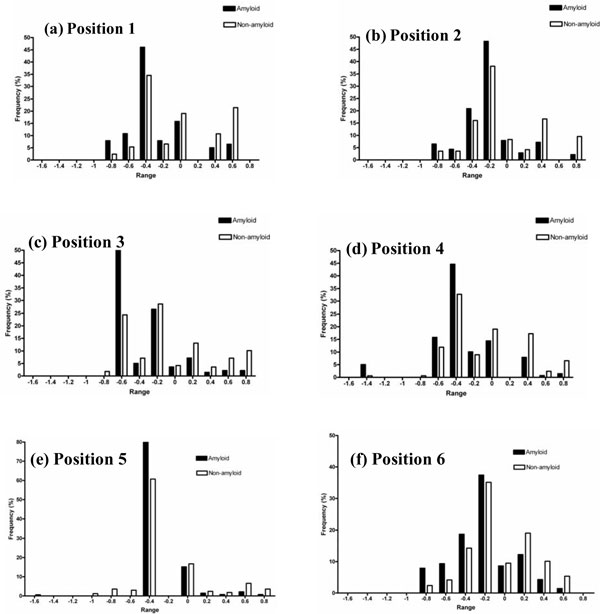
**Frequency of occurrence of amyloid and non-amyloid peptides at various ranges of potentials**.

The amyloid and non-amyloid energy potentials (ϕ(i,j) at different positions in the hexa-peptides were computed from amino acid propensities from 139 amyloid and 168 non-amyloid peptides. The next step was to compute the energy difference potentials for all the 20 amino acids and at all the six positions. The distribution of these energy differentials was analyzed for each of the six positions and the results are shown in Figure 3. Specifically, there is a marked difference between amyloids and non-amyloids at the energy cutoff of -0.2 kcal/mol. At this threshold value, 73% of peptides are amyloids and 49% are non-amyloids at position 1, 80% are amyloids and 61% are non-amyloids at position 2, 75% are amyloids and 52% are non-amyloids at position 4 and 73% are amyloids and 56% are non-amyloids at position 6. Interestingly, at position 4, 5% of the amyloids have the energy value of -1.4 kcal/mol. Further, at position 3, the amyloid and non-amyloid peptides are distinguished with 52% and 24% respectively in a narrow range of -0.6 to -0.4 kcal/mol. Similar trend is also observed at position 5 with the dominance of 80% and 61% for amyloids and non-amyloids, respectively. The general trend is the accumulation of amyloid peptides at the lower range (more stable) of potentials at each position.

### Average hydrophobicity of amyloid and non-amyloid forming hexa peptides

**Figure 4:**
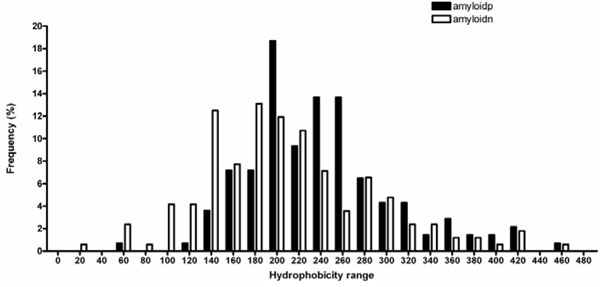
**Variation of hydrophobicity in amyloid and non-amyloid peptides**.

Hydrophobicity is an important property of peptides that from amyloid fibrils. To understand how amyloid peptides distinguish themselves than the non-amyloid peptides, we have computed the total hydrophobicity (H_T_) of each amyloid and non-amyloid peptide using the scale proposed by Mant et al. [36] (see materials and methods). The total hydrophobicity values were divided into several bins and the frequency of occurrence of peptides at different ranges of hydrophobicity is plotted in Figure 4. In general, amyloid peptides have higher (H_T_) values than non-amyloid peptides; and non-amyloid peptides are more frequent than amyloid peptides up to the H_T _value of 200. At H_T _≥ 200, the amyloid peptides are more frequent. The average H_T _values for the 139 amyloid and 168 non-amyloid peptide, are 251.1 ± 67.6, and 217.6 ± 79.0, respectively. This result is consistent with the observation of increased hydrophobicity of amyloidogenic regions in proteins [10,11,17,18].

### Variation of the 49-amino acid properties between amyloid and non-amyloid forming peptides

To uncover all features which may be different between amyloid and non-amyloid peptides, we have computed the average values of 49 diverse amino acid properties [23]. The results are summarized in Table 3. For most of the properties the differences are very small. This is expected because the two peptide sets are highly homologous. However, property numbers 4, 32, 33 and 39 show large differences between amyloid and non amyloid peptides. These properties are polarity, solvent accessible surface area for native protein and protein unfolding, and unfolding hydration heat capacity change. Interestingly, these properties refer to electrostatics, solvent accessibility as well as thermodynamics, indicating that the forces involved in protein folding and amyloidosis are common. We performed correlation and chi-square analysis between the average property values obtained with amyloid and non-amyloid peptides, and the results showed that the distributions are highly similar (r = 0.99, χ^2 ^= 1).

**Table 3:** Average values for the 49 properties used in the present study

Property	Amyloid	Non-amyloid	Difference
*K*^0^	-31.966	-31.675	-0.291
*H*_t_	1.693	1.546	0.147
*H*_p_	13.461	13.001	0.460
*P*	6.535	12.132	-5.597
p^*H*i^	5.801	5.997	-0.196
p^*K*'^	2.004	2.046	-0.042
*M*w	134.369	135.217	-0.848
*B*_l_	17.329	16.377	0.952
*R*_f_	12.353	11.152	1.202
μ	18.155	18.051	0.104
*H*_nc_	0.497	0.264	0.233
*E*_sm_	1.207	1.201	0.006
*E*_l_	0.608	0.563	0.044
*E*_t_	1.817	1.767	0.050
*P*_α_	1.032	1.050	-0.018
*P*_β_	1.231	1.088	0.143
*P*_t_	0.817	0.879	-0.062
*P*_c_	0.875	0.926	-0.051
*C*_a_	34.890	34.666	0.224
F	0.895	0.934	-0.039
*B*_r_	0.369	0.321	0.048
*R*_a_	4.998	4.409	0.589
*N*_s_	6.544	6.250	0.294
α_N_	1.111	1.079	0.032
α_C_	0.929	1.030	-0.101
α_m_	0.968	0.990	-0.022
V_0_	95.343	94.341	1.002
*N*_m_	1.818	1.848	-0.030
*N*_l_	4.497	4.179	0.318
*H*_gm_	13.845	13.459	0.386
*ASA*_D_	156.030	156.043	-0.013
*ASA*_N_	43.340	49.795	-6.455
Δ*ASA*	112.345	105.918	6.427
Δ*Gh*	-1.662	-1.903	0.241
G_hD_	-2.864	-3.460	0.596
G_hN_	-1.172	-1.455	0.283
ΔH_h_	-4.896	-4.964	0.067
-TΔ*S*_h_	3.235	3.061	0.174
Δ*C*_ph_	25.269	22.474	2.796
Δ*G*_c_	1.854	2.074	-0.220
ΔH_c_	5.833	5.733	0.100
-*T*Δ*S*_c_	-3.983	-3.663	-0.321
Δ_*G*_	0.190	0.170	0.020
Δ_*H*_	0.934	0.766	0.168
-TΔ_S_	-0.744	-0.597	-0.147
v	4.253	4.314	-0.061
s	1.382	1.460	-0.078
f	1.788	1.916	-0.128
P_ϕ-ψ_	0.817	0.955	-0.138

*Abbre*v*iations: K*^0^, compressibility; *H*_t_, thermodynamic transfer hydrophobicity; *H*_p_, surrounding hydrophobicity; *P*, polarity; p*H*i, isoelectric point; p*K*':equilibrium constant with reference to the ionization property of COOH group; *M*_w_, molecular weight; *B*_l _, bulkiness; *R*_f_, chromatographic index; β,refractive index; *H*_nc _, normalized consensus hydrophobicity; *E*_sm_, short and medium range non-bonded energy; *E*_l _, long-range non-bonded energy; *E*_t_, total non-bonded energy; *P*_α_, *P*_β_, *P*_t _and *P*_c _are, respectively, β-helical, β-structure, turn and coil tendencies; *C*_a_, helical contact area; *F*, mean rms fluctuational displacement; *B*_r_, buriedness; *R*_a_, solvent accessible reduction ratio; *N*_s _, average number of surrounding residues; β_N_, β_C _and β_m _are, respectively, power to be at the N-terminal, C-terminal and middle of β-helix; *V*^0^, partial-specific volume; *N*_m _and *N*_l _are, respectively, average medium and long-range contacts; *H*_gm_, combined surrounding hydrophobicity (globular and membrane); *ASA*_D_, *ASA*_N _and β*ASA *are, respectively, solvent accessible surface area for denatured, native and unfolding; β*G*_h _, *G*_hD _and *G*_hN _are, respectively, Gibbs free energy change of hydration for unfolding, denatured and native protein; β*H*_h_, unfolding enthalpy change of hydration; -*T*β*S*_h_, unfolding entropy change of hydration; β*C*_ph _, unfolding hydration heat capacity change; β*G*_c _, β*H*_c _and -*T*β*S*_c _are, respectively, unfolding Gibbs free energy, unfolding enthalpy and unfolding entropy changes of chain; β*G*, β*H *and -*T*β*S *are, respectively, unfolding Gibbs free energy change, unfolding enthalpy change and unfolding entropy change; *v*, volume (number of non-hydrogen side chain atoms); *s*, shape (position of branch point in a side-chain); *f*, flexibility (number of side-chain dihedral angles); *P*_ϕ-ψ_: backbone dihedral probability.

### Discrimination between amyloid and non-amyloid forming peptides

Can the above described position specific sequence features distinguish amyloid-fibril forming peptides from their close homologues that do not from the amyloid-fibrils? We have made an attempt to discriminate amyloid and non-amyloid forming peptides using hydrophobicity, 49 different properties and the energy potentials. Discrimination based on the energy differentials performed better than the other properties. We devised a statistical method to discriminate amyloid and non-amyloid peptides using total potential computed with Eqn. 6. For each peptide, k, the total energy ϕ(k) was computed for both amyloid-fibril formation and not (from non-amyloid potential). The difference between the energy potentials yield the discriminator, Δϕ (k) (see eqn. 7) The results showed that amyloid peptides are well discriminated with an accuracy of 89% (123/139). This value compares favorably with other prediction methods [10,20,25]. However, the discriminator yielded only marginal performance for non-amyloid peptides (accuracy: 54%). Taken together, these results indicate that for a given short peptide sequence, the prediction that it will form amyloid fibrils is easier to make than otherwise. That is, it is harder to predict that the peptide will not form amyloid fibrils. These and other aspects of our work are discussed in the "Discussion" section.

### Use of machine learning techniques for discrimination between amyloid and non-amyloid peptides

We have utilized several machine learning techniques for discriminating between amyloid and non-amyloid peptides as described in the Methods section. Overall, most algorithms showed similar performance and the method based on Random forest performed the best. In a 10-fold cross-validation exercise, this method yielded an accuracy of 82.1% when the statistically derived position-specific energy potentials were used. The sensitivity and specificity are 79.9% and 83.9%, respectively. Combining these energy potentials with three amino acid properties, hydrophobicity, isoelectric point and long-range non-bonded energy improved the accuracy marginally to 82.7% (sensitivity, 81.3%; specificity, 83.9%). The method was also tested with 5-fold and 20-fold cross-validations and the accuracies are 80% and 81.1%, respectively.

## Discussion

Fibril forming portions of many amyloidogenic proteins have been traced to short peptides by many experimental groups. The smallest length for a peptide that forms amyloid fibrils is three amino acid residues[43]. Tetra-peptides have also been shown to form amyloid-fibrils [12]. The most common sequence lengths for the amyloid-fibril peptides are 5-9 residues. We chose to focus on hexa-peptides because hexa-peptides have been often used in experiments to grow amyloid-fibrils [10,11]. Available experimental data on short peptides that form amyloid fibrils and those that do not form amyloid fibrils shows that even single residue differences are important [10,11]. With the growing interest in nano-materials made out of peptide aggregates with well-defined fibrillar morphologies and desirable properties [44], it has become important to computationally predict which of the short peptide sequences are capable of forming amyloid fibrils with desired properties and which of them would not yield such fibrils, even though they may form amorphous β-aggregates and may still contain kernels of the cross-β steric zipper motif. To our knowledge, this is the first attempt to discriminate between amyloid fibril and non-amyloid fibril forming peptide sequences using empirical/computational means. Here, we have used publicly available information on Amyl Hex and other hexa-peptides to uncover the subtle sequence-structural features that could be different between amyloid and non-amyloid peptides. The sequences in the two peptide datasets are highly homologous and almost all non-amyloid peptides do form amorphous β-aggregates [10]. Thus, it was not surprising that almost all of the 49 physico-chemical amino acid properties [23] showed only small differences between amyloid and non-amyloid peptide sets. Despite the high sequence homologies, the overall and position-wise amino acid propensities are different between amyloid and non-amyloid peptide sets. This indicates that amino acid side chains do play a role in amyloid-fibril formation even though the process has been thought to be mainly driven by backbone [41]. The differences in sequence features and positional context in formation of amyloid and non-amyloid peptides were converted in to the energy potentials in this study. These potentials were able to successfully identify the amyloid-peptides in most cases. This validates our approach for predicting potential amyloidogenic sequences.

Almost all studies in computational biology focus on making positive predictions. However, in this study we attempted to make negative predictions also. That is, we tried to predict that a given peptide *will not *from amyloid fibrils, even though it may self-associate via cross-β motif and form amorphous β-aggregates. In this case, making the negative prediction proved to be harder than the positive prediction that a peptide *will *form amyloid-fibril. There could be several reasons for this. First of all, kinetics of amyloid fibril formation depend on critical monomer concentrations required to initiate the process [45]. The critical monomer concentrations required for initiation of fibril formation were found to vary in the range of 30-400 μM for highly homologous tetra-peptides, KFFE, KVVE, KLLE [12,44]. However, the experiments that determine which peptides in a given set form amyloid fibrils use a single concentration value for all the peptides. The time periods over which fibril are grown are also arbitrarily set (see method section in [10-12]). These experimental condition requirements imply that the peptides which require higher critical monomer concentrations to initiate fibril formation and/or which have slower fibril growth kinetics may be falsely designated as non-amyloid peptides even-though their sequences may contain all the required features for amyloid-fibril formation. Secondly, the preferences for the individual amino acid residues to occur at each of the six positions are better characterized for amyloid and non-amyloid peptides than avoidances (Table 2), that is, amyloid fibril forming sequence features are based on positive, and not negative, selection of the relevant physicochemical properties at the level of individual amino acids. Third, the peptide sequences in the two data set are highly homologous and most of the peptides in the non-amyloid set, derived from the parent amyloid-fibril forming peptide sequence, STVIIE [10] form β-aggregates. It is quite probable that many of the peptides in non-amyloid set would form amyloid-fibrils in slightly different experimental conditions.

The prediction for non-amyloid peptides improved when three amino acid properties, namely, hydrophobicity, isoelectric point and long range interaction energy, were combined with the position specific energy potentials and machine learning techniques were used. In such techniques, the data are trained so that the methods should perform equally well for both amyloids and non-amyloid peptide sets. Not surprisingly, this procedure showed similar levels of sensitivity and specificity, and the accuracy is the balance between these two terms.

## Conclusions

We have analyzed the available experimental data on the hexa-peptides that from amyloid fibrils and on those that do not form amyloid-fibrils. We found that amyloid peptides show position-specific preference and avoidances that are different from their homologues which may form β-aggregates but not fibrils. These position-specific preferences of amino acid residues have been utilized to discriminate amyloid forming peptides and non-amyloids using statistical methods and machine learning techniques. In the next step, we plan to combine single residue propensities with the residue pair propensities in a position wise manner to further improve our ability to predict both amyloid and non-amyloid forming hexa-peptides.

## Authors' contributions

MMG, SK and DV conceived the project. AMT and MMG carried out the computations. AMT, SK, DV and MMG contributed in discussions and preparing the manuscript. All authors read and finalized the manuscript.

## Competing interests

The authors declare that they have no competing interests.

## Declaration

The publication charges are funded by the Indian Institute of Technology Madras.

This article has been published as part of *BMC Bioinformatics *Volume 14 Supplement 8, 2013: Proceedings of the 2012 International Conference on Intelligent Computing (ICIC 2012). The full contents of the supplement are available online at http://www.biomedcentral.com/bmcbioinformatics/supplements/14/S8.
